# Sequential induction chemotherapy followed by radical chemo-radiation in the treatment of locoregionally advanced head-and-neck cancer

**DOI:** 10.1038/sj.bjc.6604444

**Published:** 2008-06-17

**Authors:** S A Bhide, M Ahmed, Y Barbachano, K Newbold, K J Harrington, C M Nutting

**Affiliations:** 1 Head and Neck Unit, Royal Marsden Hospital, London SW3 6JJ, UK; 2 The Institute of Cancer Research, 237 Fulham Road, London SW3 6JB, UK; 3 Department of Statistics, Royal Marsden Hospital, Sutton, Surrey, SM2 5PT

**Keywords:** induction, chemotherapy, chemo-radiation, advanced, head-and-neck cancer

## Abstract

We describe a retrospective series of patients with advanced head-and-neck cancer who were treated with induction chemotherapy followed by radical chemo-radiation. Patients treated with two cycles of induction chemotherapy followed by definitive chemo-radiation for squamous cell carcinoma of the head-and-neck region, from 2001 – 2006 at the Royal Marsden Hospital, formed the basis of this study. Cisplatin (75 mg m^−2^) on day 1 and 5-FU (1000 mg m^−2^) day 1 – 4 was the standard regimen used for induction treatment. Cisplatin (100 mg m^−2^) on day 1 and day 29 was used for concomitant treatment. The radiation was delivered using conformal technique. Tissues containing macroscopic and microscopic disease were treated to doses of 65 Gray (Gy) in 30 fractions and 50 Gy in 25 fractions, respectively. Data on patterns of relapse and acute toxicity (NCICTCv.3.0) were collected. A total of 129 patients were included, median age was 58 (range: 27 – 78). The site of tumour was: oropharynx 70 (54%), larynx 30 (23%), hypopharynx 24 (19%) and other 5 (4%). The median follow-up was 19 months (range: 4 – 58). Local control, disease-specific survival and overall survival at 2 years were 71%, 68% and 63%, respectively. The distant recurrence rate at 2 years was 9%. Ten patients required dose reduction during induction chemotherapy due to toxicity. The dose of 5-FU was reduced in six patients and that of cisplatin in four patients. The incidence of grade 3/4 toxicity was: neutropenia 5%, thrombocytopenia 1%, nausea and vomiting 3%. One cycle of concurrent cisplatin was omitted in 23 patients due to toxicity. Full-dose radiotherapy was administered to 98% of patients. The incidence of grade 3/4 toxicity was: skin 20%, dysphagia 65%, mucositis 60%, neutropenia 3%, anaemia 1%, nausea and vomiting 4%, nephrotoxicity 1%. Induction chemotherapy followed by radical chemo-radiation is a safe and tolerable regimen in the treatment of advanced head-and-neck cancer. Distant recurrence rates are lower with equivalent local control and survival compared to chemo-radiation alone (historical controls).

Surgery and radiotherapy are the mainstays of treatment for head-and-neck cancer patients. However, in recent years, systemic chemotherapy has been increasingly incorporated into the treatment plan in patients with stages III and IV disease. As part of the primary treatment, systemic chemotherapy can be administered before (induction chemotherapy), or during radiotherapy (concomitant chemotherapy). Meta-analysis showed that concurrent chemotherapy confers an absolute survival benefit of 8% (Pignon *et al*, 2000). Concurrent chemo-radiation is the current standard of care as the first line treatment for stages III and IV disease. Two recent trials have also reported on the benefit of concomitant chemo-radiotherapy in the post-operative setting for high-risk patients (Bernier *et al*, 2005). In contrast, the role of induction chemotherapy is not yet clear. Although not the standard of care, induction chemotherapy is used in clinical practice and is thought to be beneficial for reducing the rate of distant metastases (Paccagnella *et al*, 1994; Lefebvre *et al*, 1996), increasing organ preservation (Group, 1991; Lefebvre *et al*, 1996) and survival rates (Paccagnella *et al*, 1994; Zorat *et al*, 2004). Combining induction and concomitant chemotherapy with radiation, that is, in a sequential treatment approach, has the potential for improving disease outcomes.

A combination of cisplatin (75 – 100 mg m^−2^) and 5-fluorouracil (5-FU, 750 – 1000 mg m^−2^ for 5 days) every three weeks is the most commonly used regimen for induction treatment. Single-agent cisplatin is the cytotoxic agent of choice for concomitant chemo-radiotherapy. The majority of clinical trials reported in the literature have not used these standard aforementioned regimens.

At our institution, two cycles of induction cisplatin (75 mg m^−2^) plus 5-FU (1000 mg m^−2^ for 4 days) followed by radical chemo-radiation with two cycles of cisplatin (100 mg m^−2^) has been the standard of care for patients with stages III and IV head-and-neck cancer since 2001. This regimen was initiated in response to a desire to deliver short-course induction chemotherapy during the waiting period (average 4 weeks in 2001) for planning and commencing radiotherapy. The relatively conservative dosing of cisplatin (75 rather than 100 mg m^−2^) and 5-FU (4 rather than 5 days) was an attempt to limit the risk that chemotherapy-induced toxicity might delay the start of chemo-radiotherapy, which is considered to be the most important curative component of the treatment approach. Similarly, the decision to restrict induction chemotherapy to two, rather than three or four, cycles was motivated by the wish to commence chemo-radiotherapy as soon as possible after the planning process was complete. In this report, we present data on the toxicity and outcomes for the retrospective study performed using this regimen in advanced head-and-neck cancer.

## Materials and methods

From January 2001 to September 2006, 145 consecutive patients with head-and-neck cancer were treated at the Royal Marsden Hospital with two cycles of induction chemotherapy followed by radical chemo-radiation (CRT). This report provides data on patients with an established primary tumour of squamous cell (SCC) histology. Patients who presented with carcinoma of the nasopharynx (regardless of WHO type), with WHO performance status two and above, or a tumour of unknown primary origin are not included in this report. All patients were reviewed in a multidisciplinary team meeting before the start of treatment. Our institutional policy is to offer organ-preserving treatment to all patients with locally advanced head-and neck-cancers. Therefore, specific data on whether, or not, a tumour was technically operable at diagnosis were not available.

### Chemotherapy schedule

The induction chemotherapy regimen for locally advanced head-and-neck cancer was two cycles of platinum-based chemotherapy followed by 4 days of 5-FU given on a 21-day cycle. The majority of patients received cisplatin chemotherapy 75 mg m^−2^ day 1 with 5-FU 1g m^−2^ day 1 – 4. This dose of cisplatin was selected in an attempt to limit adverse effects in our unselected population of patients and to maximise the number of patients who were able to receive cisplatin during concomitant CRT. Patients who had poor renal function at the outset (glomerular filtration rate of <60 ml per min^−1^) received carboplatin-5 fluorouracil and concomitant carboplatin. These patients were excluded from the analysis. Concomitant cisplatin (100 mg m^−2^ on days 1 and 29) was administered as two, rather than three, cycles to allow us to employ our standard modestly accelerated hypofractionated radiotherapy regimen.

### Radiation delivery

All patients received a radiobiological equivalent dose of 70 Gy in 35 fractions to the tumour and high-risk nodal groups. The majority of patients were treated using a radiotherapy fractionation of 65 Gy in 30 fractions over 6 weeks. The last week of treatment was given as an accelerated hypofractionated course of 15 Gy in five fractions. Elective nodes received 50 Gy in 25 fractions at conventional fractionation. During the radiotherapy, concomitant cisplatin was given at 100 mg m^−2^ on days 1 and 29. All patients receiving other fractionation regimens were included, provided they were planned to receive a biologically equivalent dose (BED) equal to or greater than our standard fractionation. Patients who were planned with 2D, 3D conformal or intensity-modulated radiotherapy were included in the analysis, provided they were planned to receive both induction and concomitant chemotherapy. Patients who had residual disease at the end of their course of treatment were referred for salvage surgery, provided their disease was operable. Patients who present with node-positive disease underwent a neck dissection after chemo-radiation, unless they achieved complete response (CR) as per the departmental policy.

### Outcome measures

Data on patient demographics and stage were collected. Patients were staged using radiology (CT and/or MRI) and an examination under anaesthesia and biopsy. Response rates to induction chemotherapy and to CRT were assessed clinically and radiologically. Radiological responses were recorded according to RECIST criteria. A patient was deemed to have had a CR, if there was no evidence of disease either clinically or on imaging following CRT (and salvage surgery when required). Time to disease progression was measured form the date of diagnosis to the time of first recurrence. Survival was measured form the date of diagnosis to the date of death. Toxicities for induction and CRT were recorded and graded from 1 – 4 according to the Common Terminology Criteria for Adverse Events v3.0 (CTCAE) (DCTD, 2003). Patients were not reviewed weekly during induction chemotherapy and blood tests were limited to occasions when the patients were attending the hospital for radiotherapy planning appointments. Therefore, it is likely that the documented haematological and biochemical toxicities may be an underestimate of the true values. During chemo-radiotherapy, patients were reviewed weekly and underwent weekly blood tests. Routine baseline audiometry was not performed and evaluation of ototoxicity was based on clinical assessment.

### Statistical analysis

Local control, disease-specific and overall survival rates at 2 years were obtained using Kaplan—Meier survival curves. Data for patients lost to follow-up or whose follow-up did not reach 2 years was censored. Local control rate was defined as the proportion of patients who were free of local recurrence at 2 years. Disease-specific survival rate defined the percentage of patients who were free of their head-and-neck cancer at 2 years. Univariate analyses were performed for gender, T and N stages, age, smoking status, alcohol ingestion, site and stage of disease followed by a multivariate analysis.

## Results

Of the initial 145 consecutive patients, 129 are included in the final analysis. Sixteen patients who had received carboplatin throughout their treatment were excluded from analysis. The patient demographics and TNM staging are listed in Tables 1 and 2, respectively. The median follow-up was 19 months (range: 4 – 58). The majority of patients were found to have stage III/IV tumours. Two patients had stage II tumours (T2N0M0) and one stage I. One of these patients had a bulky transglottic laryngeal carcinoma, while the other had a bulky T2 tonsillar tumour extending to midline.

### Toxicity

A total of 119 patients completed full-dose induction chemotherapy. Ten patients required dose reduction during induction chemotherapy due to toxicity. One patient had grade 3 diarrhoea, three patients had grade 4 haematological toxicity, two patients had dose-limiting renal toxicity and four had dose-limiting cardiac toxicity. Four more patients had cardiac toxicity during induction chemotherapy, which was not considered to be dose limiting. Of the eight patients who developed cardiac toxicity, three developed atrial fibrillation and five developed coronary vasospasm and chest pain. The dose of 5-FU was reduced in six patients and that of cisplatin in four patients. The incidence of grade 3/4 toxicity was: neutropenia 5%, thrombocytopenia 1%, nausea and vomiting 3% (Table 3). Overall, induction chemotherapy in this group of patients was tolerated well. A total of 106 patients (82%) completed the full course of concomitant cisplatin. In the remaining patients, cisplatin was replaced by carboplatin (AUC5) for the second cycle due to toxicity. One hundred and twenty-seven (98%) patients completed the full course of radiotherapy. One patient missed the last two fractions due to radiation-induced laryngeal oedema. The second patient missed the last six fractions of treatment due to upper gastrointestinal bleed from a duodenal ulcer. The incidence of grade 3 toxicity was: skin 26%, dysphagia 85%, mucositis 78%, neutropenia 3%, anaemia 1%, nausea and vomiting 4% and nephrotoxicity 1% (Table 3). No grade 4 toxicity was seen during chemo-radiation. There were no deaths related to toxicity during induction chemotherapy and chemo-radiation.

Ten patients developed grade 3/4 late radiation toxicity at 1 year. Two patients had osteoradionecrosis of the mandible; seven patients had persistent dysphagia from abnormal anterior laryngeal movement and narrowing at the cricopharyngeal inlet and one patient had laryngeal necrosis. Three patients required laryngo-pharyngectomy for late radiation toxicity and one patient was successfully treated with oesophageal dilatation.

### Response rate

At the end of two cycles of induction chemotherapy, seven (5.4%) achieved radiological CR, 92 (71.3%) patients achieved partial response (PR) and 30 (23%) patients had stable disease (SD). At the end of CRT, residual disease remained in 13 of 92 (14.1%) patients who had had a PR and 14 of 30 (46.7%) patients who had had SD. A total of 102 (79.1%) patients achieved a CR; residual disease was present at the end of treatment in 27 patients. Of these 27 patients, 18 had biopsy-proven residual disease at the end of treatment. Ten patients underwent salvage surgery. Out of the remaining 17 patients, 12 remained inoperable at the end of treatment. Only one patient was operable at initial diagnosis. Two patients refused salvage surgery and two patients were considered to be medically unfit for surgery.

### Local control rate

**Figure 1:**
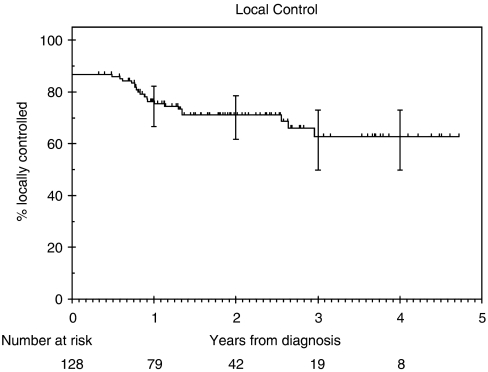
Kaplan—Meier estimates of local control.

The loco-regional control rates were 75% (95% CI: 67 – 82%) and 71% (95% CI: 62 – 79%) at 1 year and 2 years, respectively (Figure 1). Among the patients with laryngeal/hypopharyngeal cancer, 13 (24%) developed local recurrence, one of whom had a nodal recurrence. Six patients underwent salvage laryngo-pharyngectomy, one patient refused surgery and the remaining patients were considered inoperable. The laryngeal preservation rate (for patients with laryngeal/hypopharyngeal cancer) at 2 years was 62%. Eight (9%) out the 92 patients who had N+ disease at presentation had nodal recurrence. Two underwent neck dissection and six were considered to be inoperable. Various factors analysed by using univariate analysis are listed in Table 4. T stage (*P*=0.009) remained the only significant factor on multivariate analysis.

### Survival

**Figure 2:**
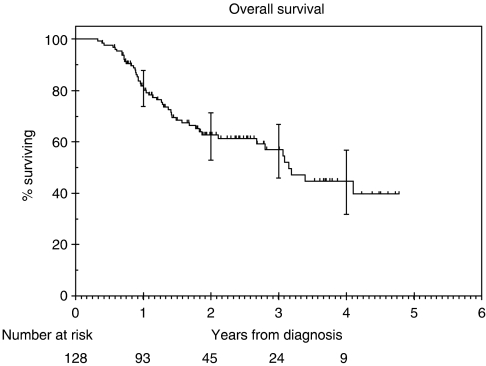
Kaplan—Meier estimates of overall survival.

The overall survival at 2 years was 63% (95% CI: 53 – 71%). Overall survival is demonstrated in Figure 2. Various factors analysed by using univariate analysis are listed in Table 4. T (*P*=0.001) and N stages (*P*=0.018) were significant factors on univariate analysis. However, on multivariate analysis, the only factor affecting overall survival was T stage (*P*<0.001). N stage was not significant on multivariate analysis (*P*=0.06). When nodal status was broken down according to N0/N1 or N2/N3, it was still not significantly associated with survival on multivariate analysis (*P*=0.06), The median survival for all patients was 3.2 years (95% CI: 2.6 – 3.7).

**Figure 3:**
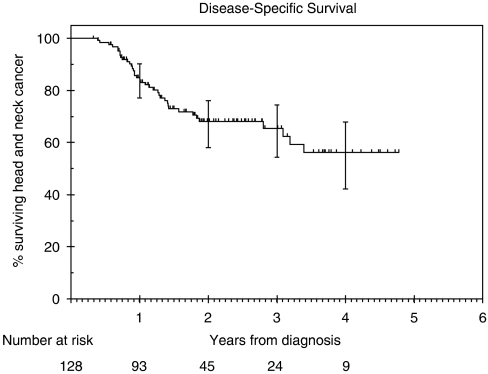
Kaplan—Meier estimates of disease-free survival.

The disease-specific survival (DSS) at 2 years was 68% (95% CI: 58 – 76%). At the time of the analysis, median DSS was not reached (Figure 3). Various factors analysed using univariate analysis are listed in table, only T (*P*=0.005) and N stages (*P*=0.007) were significant. On multivariate analysis, these factors remained significant (T stage (*P*=0.035), N stage (*P*=0.029)). Of the patients who died from head-and-neck cancer, 84% died due to uncontrolled or relapsed loco-regional disease.

The rate of freedom from distant metastases was 93 and 91% at 1 and 2 years, respectively. There were 12 deaths from intercurrent illnesses: nine patients (7%) died from a second primary cancer (six non-small cell lung cancer, two oesophageal cancer, one breast cancer); one patient died of respiratory infection; one patient died of cerebrovascular disease and one patient died of myocardial infarction.

## Discussion

The patients in our study had a 2-year local control rate of 71%, DSS of 68%, overall survival of 63%. We identified only one study in the literature using a treatment regimen similar to ours (Psyrri *et al*, 2004). Psyrri *et al* (2004) reported on a phase II study with 42 patients with stages III and IV head-and-neck cancer patients. At 2 years, the authors reported a local control rate of 76.3%, DSS of 69%, overall survival of 66.7% and rate of control of distant metastases of 79%. This study included patients with nasopharyngeal tumours, which have a better stage for stage outcome compared to tumours at other head-and-neck sites. This might account for the slightly inferior outcomes in our study.

Two other studies using cisplatin and 5-FU for induction and cisplatin alone for concomitant treatment have been reported in the literature. The study by Hitt *et al*, 2005 (189 patients) used three cycles of induction and concomitant chemotherapy. This was compared in a randomised setting to an induction regimen that included paclitaxel. The SWOG study (42 patients) used two cycles of induction treatment but three cycles of concomitant chemotherapy (Urba *et al*, 2005). The overall survival at 2 years in the Hitt study was 53.6% in the control arm (without paclitaxel). The overall survival in the SWOG study at 3 years was 64%. The superior results in the SWOG study might be due to the exclusion of patients with T4 tumours. The rates of local control or the DSS were not stated in the published reports of these studies.

Chemo-radiation is the standard treatment for patients with advanced head-and-neck cancer. Table 5 summarizes the disease outcomes from a few published trials using chemo-radiation for treatment of advanced head-and-neck cancer. The 2-year loco-regional control rate (71%) and overall survival (63%) in our study was superior to the studies with reported 2-year outcomes. This could be due to a combination of low nodal recurrence rates (9%) in patients with N+ disease and reduced rate of distant metastases. This is consistent with the Pignon meta-analyses where the analyses of trials using the cisplatin and 5-FU regimen for induction treatment showed an absolute survival benefit of 5% (Pignon *et al*, 2000).

Organ conservation is one of the rationales for induction chemotherapy (Group, 1991; Lefebvre *et al*, 1996). In our study, the laryngeal preservation rate at 2 years was 62%. This included patients who had died without loco-regional recurrence and therefore had an intact larynx at the time of death. This rate is comparable to the other studies. In the phase II studies, using the sequential approach by Psyrri *et al* (Psyrri *et al*, 2004) and the SWOG study (Urba *et al*, 2005), the larynx preservation rates were 64.3 and 64%, respectively.

It has been postulated that the induction chemotherapy reduces the rates of distant metastases (Paccagnella *et al*, 1994; Lefebvre *et al*, 1996). In our study, the rate of freedom from distant metastases was 91% at two years. This is comparable to other studies using the sequential approach: Psyrri *et al* (2004; 79%), Machtay *et al* (84%) and Vokes *et al* (93%). It is better than the rate of distant metastases in trials using chemo-radiation alone (see Table 5).

Treatment-related toxicity in our study was not excessive. A total of 90% of the patients completed full-dose induction treatment and 82% of the patients had full-dose concomitant cisplatin. None of the patients required treatment gaps during chemo-radiation due to the toxicity. Although, the incidence of acute toxicity during chemo-radiation was quite significant, it was transient and the majority of patients recovered. Enteral feeding was required in 85% of our patients due to severe mucositis. This is much higher than that reported for trials using chemo-radiation alone (33 – 44%). However, most of these patients recovered and only four patients (3%) required enteral support after 1 year. This rate is considerably lower than the rates of late feeding tube dependence (24 – 50%) in other series.

The overall survival in this group of patients leaves scope for improvement. Phase II studies have been conducted using taxanes in addition to cisplatin and 5-FU (Colevas *et al*, 1999; Posner *et al*, 2001). These have shown better response rates and tolerable toxicity levels. There have been three randomised trials adding taxanes to the standard sequential approach. In one study, paclitaxel was added to cisplatin and 5-FU in the experimental arm (Hitt *et al*, 2005). Although the response rates were better in the experimental arm, there was no significant difference in the overall survival (51 *vs* 43%, *P*=0.063). Furthermore, the design of that study incorporated a planned neck dissection between the induction chemotherapy and chemo-radiotherapy phases of treatment. In our view, this represented a suboptimal approach, since the delay for surgery and wound healing potentially provided an opportunity for repopulation at the primary tumour site. Therefore, when those data were published, we decided to continue to treat patients with our standard protocol of induction cisplatin/5-FU followed by chemoradiation. More recently, two phase II studies have been published (EORTC24971/TAX323 and TAX 324) in which docetaxel was added to cisplatin and 5-FU in the experimental arm for induction treatment (Posner *et al*, 2007; Vermorken *et al*, 2007). The study by Vermorken *et al* (2007) showed a survival benefit for the docetaxel arm but the overall 2-year survival (43%) was lower than in our study or other reported studies using the sequential approach. However, this study exclusively included patients with unresectable disease and concomitant chemotherapy was not used. Therefore, it is difficult to draw any conclusions from this study with regard to the benefit of taxanes in patients treated using the sequential approach. Posner *et al* (2007), demonstrated a statistically significant 2-year survival of 68% for the TPF arm *vs* 55% for the PF arm using the sequential approach but that study employed an unconventional concomitant regimen of carboplatin AUC 1.5 every week. It is possible that the statistically significant difference observed in the TAX 324 study could be due to a suboptimal control arm, that is, using carboplatin instead of cisplatin for concomitant treatment. This supposition is further supported by the 63% 2-year survival for PF followed by CRT in our study, which was superior to the control arm of the TAX 324 study. Currently, there are no data for a head-to-head comparison of carboplatin *vs* cisplatin in this setting, but the meta-analysis most strongly supports the use of cisplatin for concomitant treatment (Pignon *et al*, 2000). Thus, until randomised data are published from studies that employ standard cisplatin-based concomitant regimens, it is difficult to draw definitive conclusions on the utility of taxanes as part of induction chemotherapy regimens.

Patients with oropharyngeal cancers comprised the largest group (54%) in this study. This observation is important, because it is known that a subset of these tumours have a different aetiology (human papilloma virus (HPV) infection) and a better prognosis when compared with ordinary tobacco- and alcohol-induced cancers (Weinberger *et al*, 2006; Psyrri and DiMaio, 2008). However, this percentage of patients with oropharyngeal cancers is typical of other large treatment series, including the two recently published phase III trials of taxane-containing induction chemotherapy (Posner *et al*, 2007; Vermorken *et al*, 2007). In those studies, patients with oropharyngeal tumours comprised 46 and 53% of the treated subjects, respectively. Therefore, although we do not have data on the number of patients with HPV-related tumours in our series, it is likely that the rates would have been comparable to those in other series.

### CONCLUSIONS

This study shows the results of induction chemotherapy using the PF regime followed by radical CRT forms as a baseline for investigation of new treatments in advanced head-and-neck cancer. Randomised phase III trials comparing the standard sequential approach (using cisplatin and 5-FU) to concomitant chemo-radiation (using cisplatin) are required to provide a definite answers to the questions regarding the benefit of induction chemotherapy.

## Figures and Tables

**Table 1:** Patient demographics

**Number of patients**	**129**
*Sex*	
Male	78 (76%)
	
*Age(years)*	
Median (range)	58 (27 – 78)
Smokers	95 (74%)
	
*Site*	
Oropharynx	70 (54%)
Larynx	30 (23%)
Hypopharynx	24 (19%)
Oral cavity	3 (2.3%)
Paranasal sinuses	2 (1.7%)
	
*Stage*	
I	1 (0.8%)
II	2 (1.6%)
III	48 (37.2%)
IV	78 (60.5%)

**Table 2:** TNM staging distribution

**T/N Stage**	**N0**	**N1**	**N2a**	**N2b**	**N2c**	**N3**	**Total**
T1	1	0	3	1	0	0	5
T2	2	8	4	8	7	1	30
T3	21	12	4	5	9	6	57
T4	12	6	1	10	3	5	37
Total	36	26	12	24	19	12	129

**Table 3:** Toxicity during neo-adjuvant chemotherapy and CRT

	**Neo-adjuvant chemotherapy**		**CRT**	
	**Grades 1/2**	**Grades 3/4**	**Grade 1/2**	**Grade 3**
Anaemia (%)	26 (20)	0	71 (55)	2 (1.5)
Neutropenia (%)	17 (13)	7 (5)	26 (20)	4 (3)
Thrombocytopenia (%)	1 (0.8)	1 (0.8)	5 (4)	0
Ototoxicity (%)	1 (0.8)	0	0	0
Nausea and Vomiting (%)	13 (10)	4 (3)	15 (11)	5 (4)
Neurotoxicity (%)	0	0	1 (0.8)	0
Cardiac toxicity (%)	8 (6)	0	NA	NA
Nephrotoxicity (%)	5 (4)	0	8 (6)	1 (0.8)
Skin reaction (%)	NA	NA	103 (77)	26 (23)
Mucositis (%)	0	0	51 (40)	78 (60)
Dysphagia (%)	NA	NA	36 (28)	85 (72)

NA=not applicable.

**Table 4:** Univariate analysis of potential factors affecting disease outcomes

	**P-values**		
**Factor tested**	**Local control**	**Disease specific survival**	**Overall survival**
T-stage	0.001	0.005	0.001
N-stage	0.423	0.007	0.018
Gender	0.749	0.827	0.734
Site of cancer	0.110	0.549	0.702
Age (<60 *vs* ⩾60)	0.960	0.724	0.963
Stage (III and IV)	0.531	0.208	0.108

**Table 5:** Summary of published evidence using chemo-radiation for stage III/IV head-and-neck cancer

**Study**	**Number of patients**	**Radiotherapy**	**Concurrent chemotherapy**	**Local control % (years)**	**Overall survival % (years)**	**Distant metastases % (years)**	**Late toxicity (dysphagia; %)**
Calais	109	CONV (70 Gy, 2 Gy per day)	Carboplatin/5-FU	57 (3)	51 (3)	18% (3 years)	NA
Breizel	56	HART (70 Gy, 1.25 Gy b.i.d, 1 week gap)	Cisplatin/5-FU	70 (3)	55 (3)	27% (3 years)	NA
Budach	190	HART (70.6 Gy, 2 Gy per day × 2 weeks then 1.4 Gy b.i.d × 4 weeks)	Mitomycin/5-FU	57.7 (2)	48 (2)	35 (2)	50
Wendt	130	HRT (70.2 Gy, 1.8 Gy b.i.d X10 weeks, two gaps × 11 days)	Cisplatin/5-FU	56 (3)	49 (3)	9 (3)	NA
Huguenin	112	HART (74.4 Gy, 1.2 Gy b.i.d × 6 weeks)	Cisplatin	55 (2.5)	59 (2.5)	39 (5)	24
Staar	113	C-boost (69.9 Gy, 1.8 Gy per day+1.5 Gy per day × 2 weeks)	Carboplatin/5-FU	51 (2)	48 (2)	18 (2)	30

C-boost=concomitant boost radiotherapy; CONV=conventional fractionation; HART=hyperfractionated accelerated radiotherapy; HRT=hyperfractionated radiotherapy.

## References

[bib1] Bernier J, Cooper JS, Pajak TF, van Glabbeke M, Bourhis J, Forastiere A, Ozsahin EM, Jacobs JR, Jassem J, Ang KK, Lefebvre JL (2005) Defining risk levels in locally advanced head and neck cancers: a comparative analysis of concurrent postoperative radiation plus chemotherapy trials of the EORTC (no.22931) and RTOG (no.; 9501). Head Neck 27: 843–850 16161069 10.1002/hed.20279

[bib2] Colevas AD, Norris CM, Tishler RB, Fried MP, Gomolin HI, Amrein P, Nixon A, Lamb C, Costello R, Barton J, Read R, Adak S, Posner MR (1999) Phase II trial of docetaxel, cisplatin, fluorouracil, and leucovorin as induction for squamous cell carcinoma of the head and neck. J Clin Oncol 17: 3503–3511 10550148 10.1200/JCO.1999.17.11.3503

[bib3] DCTD, N., NIH, DHHS. (2003) Common Terminology Criteria for Adverse Events, Version 3.0 ( http://ctep.cancer.gov )

[bib4] Group, T.D.o.V.A.L.C.S. (1991) Induction chemotherapy plus radiation compared with surgery plus radiation in patients with advanced laryngeal cancer. The Department of Veterans Affairs Laryngeal Cancer Study Group. N Engl J Med 324: 1685–1690 2034244 10.1056/NEJM199106133242402

[bib5] Hitt R, Lopez-Pousa A, Martinez-Trufero J, Escrig V, Carles J, Rizo A, Isla D, Vega ME, Marti JL, Lobo F, Pastor P, Valenti V, Belon J, Sanchez MA, Chaib C, Pallares C, Anton A, Cervantes A, Paz-Ares L, Cortes-Funes H (2005) Phase III study comparing cisplatin plus fluorouracil to paclitaxel, cisplatin, and fluorouracil induction chemotherapy followed by chemoradiotherapy in locally advanced head and neck cancer. J Clin Oncol 23: 8636–8645 16275937 10.1200/JCO.2004.00.1990

[bib6] Lefebvre JL, Chevalier D, Luboinski B, Kirkpatrick A, Collette L, Sahmoud T (1996) Larynx preservation in pyriform sinus cancer: preliminary results of a European Organization for Research and Treatment of Cancer phase III trial. EORTC Head and Neck Cancer Cooperative Group. J Natl Cancer Inst 88: 890–899 8656441 10.1093/jnci/88.13.890

[bib7] Machtay M, Rosenthal DJ, Hershock D, Jones H, Williamson S, Greenberg MJ, Weinstein GS, Aviles VM, Chalian AA, Weber RS (2002) Penn Cancer Center Clinical Trials Group. Organ preservation therapy using induction plus concurrent chemoradiation for advanced resectable oropharyngeal carcinoma: a University of Pennsylvania Phase II trial. J Clin. Oncol 20: 3964–3971 12351593 10.1200/JCO.2002.11.026

[bib8] Paccagnella A, Orlando A, Marchiori C, Zorat PL, Cavaniglia G, Sileni VC, Jirillo A, Tomio L, Fila G, Fede A, Endrizzi L, Bari M, Sampognaro E, Balli M, Gava A, Pappagallo GL, Fiorentino MV (1994) Phase III trial of initial chemotherapy in stage III or IV head and neck cancers: a study by the Gruppo di Studio sui Tumori della Testa e del Collo. J Natl Cancer Inst 86: 265–272 8158680 10.1093/jnci/86.4.265

[bib9] Pignon JP, Bourhis J, Domenge C, Designe L (2000) Chemotherapy added to locoregional treatment for head and neck squamous-cell carcinoma: three meta-analyses of updated individual data. MACH-NC Collaborative Group. Meta-Analysis of Chemotherapy on Head and Neck Cancer. Lancet 355: 949–955 10768432

[bib10] Posner MR, Glisson B, Frenette G, Al-Sarraf M, Colevas AD, Norris CM, Seroskie JD, Shin DM, Olivares R, Garay CA (2001) Multicenter phase I-II trial of docetaxel, cisplatin, and fluorouracil induction chemotherapy for patients with locally advanced squamous cell cancer of the head and neck. J Clin Oncol 19: 1096–1104 11181674 10.1200/JCO.2001.19.4.1096

[bib11] Posner MR, Hershock DM, Blajman CR, Mickiewicz E, Winquist E, Gorbounova V, Tjulandin S, Shin DM, Cullen K, Ervin TJ, Murphy BA, Raez LE, Cohen RB, Spaulding M, Tishler RB, Roth B, Viroglio Rdel C, Venkatesan V, Romanov I, Agarwala S, Harter KW, Dugan M, Cmelak A, Markoe AM, Read PW, Steinbrenner L, Colevas AD, Norris Jr CM, Haddad RI (2007) Cisplatin and fluorouracil alone or with docetaxel in head and neck cancer. N Engl J Med 357: 1705–1715 17960013 10.1056/NEJMoa070956

[bib12] Psyrri A, DiMaio D (2008) Human papillomavirus in cervical and head-and-neck cancer. Nat Clin Pract Oncol 5: 24–31 18097454 10.1038/ncponc0984

[bib13] Psyrri A, Kwong M, DiStasio S, Lekakis L, Kassar M, Sasaki C, Wilson LD, Haffty BG, Son YH, Ross DA, Weinberger PM, Chung GG, Zelterman D, Burtness BA, Cooper DL (2004) Cisplatin, fluorouracil, and leucovorin induction chemotherapy followed by concurrent cisplatin chemoradiotherapy for organ preservation and cure in patients with advanced head and neck cancer: long-term follow-up. J Clin Oncol 22: 3061–3069 15284256 10.1200/JCO.2004.01.108

[bib14] Urba SG, Moon J, Giri PG, Adelstein DJ, Hanna E, Yoo GH, Leblanc M, Ensley JF, Schuller DE (2005) Organ preservation for advanced resectable cancer of the base of tongue and hypopharynx: a Southwest Oncology Group Trial. J Clin Oncol 23: 88–95 15625363 10.1200/JCO.2005.04.017

[bib15] Vermorken JB, Remenar E, van Herpen C, Gorlia T, Mesia R, Degardin M, Stewart JS, Jelic S, Betka J, Preiss JH, van den Weyngaert D, Awada A, Cupissol D, Kienzer HR, Rey A, Desaunois I, Bernier J, Lefebvre JL (2007) Cisplatin, fluorouracil, and docetaxel in unresectable head and neck cancer. N Engl J Med 357: 1695–1704 17960012 10.1056/NEJMoa071028

[bib16] Vokes EE, Stenson K, Rosen FR, Kies MS, Rademaker AW, Witt ME, Brockstein BE, List MA, Fung BB, Portugal L, Mittal BB, Pelzer H, Weichselbaum RR, Haraf DJ (2003) Weekly carboplatin and paclitaxel followed by concomitant paclitaxel, fluorouracil, and hydroxyurea chemoradiotherapy: curative and organ-preserving therapy for advanced head and neck cancer. J Clin. Oncol 21: 320–326 12525525 10.1200/JCO.2003.06.006

[bib17] Weinberger PM, Yu Z, Haffty BG, Kowalski D, Harigopal M, Brandsma J, Sasaki C, Joe J, Camp RL, Rimm DL, Psyrri A (2006) Molecular classification identifies a subset of human papillomavirus-associated oropharyngeal cancers with favorable prognosis. J Clin Oncol 24: 736–747 16401683 10.1200/JCO.2004.00.3335

[bib18] Zorat PL, Paccagnella A, Cavaniglia G, Loreggian L, Gava A, Mione CA, Boldrin F, Marchiori C, Lunghi F, Fede A, Bordin A, Da Mosto MC, Sileni VC, Orlando A, Jirillo A, Tomio L, Pappagallo GL, Ghi MG (2004) Randomized phase III trial of neoadjuvant chemotherapy in head and neck cancer: 10-year follow-up. J Natl Cancer Inst 96: 1714–1717 15547184 10.1093/jnci/djh306

